# Advanced removal of phosphorus from urban sewage using chemical precipitation by Fe-Al composite coagulants

**DOI:** 10.1038/s41598-024-55713-2

**Published:** 2024-02-28

**Authors:** Hongbin Xu, Songyu Wei, Guoqiang Li, Baolei Guo

**Affiliations:** 1 School of Ecology and Environment, Zhengzhou University, Zhengzhou, 450001 China; 2 School of Water Conservancy and Transportation, Zhengzhou University, Zhengzhou, 450001 China

**Keywords:** Fe-Al composite coagulant, Urban sewage, Advanced phosphorus removal, Phosphorus form, Pollution remediation, Environmental sciences

## Abstract

Phosphorus (P) removal is a significant issue in wastewater treatment. This study applies Fe-Al composite coagulant to the advanced treatment of different P forms in biological effluent. For 90% total P removal, the dosage of FeCl_3_-AlCl_3_ composite coagulant reduces by 27.19% and 43.28% than FeCl_3_ and AlCl_3_ only, respectively. Changes in effluent P forms could explain the phenomenon of composite coagulant dosage reduction. The suspended P in the effluent of composite coagulant is easier removed by precipitation than single coagulant. In this study, the hydrolysis speciations of Fe^3+^, Fe^2+^, and Al^3+^ at a pH range are calculated by Visual MINTEQ. Changes in the morphology of metal hydroxides correlate with P removal at pH 4–9. Besides, analyses of scanning electron microscope (SEM), Fourier transformed infrared (FTIR), and X-ray photoelectron spectroscopy (XPS) are performed on the coagulation precipitations. Fe^2+^ reacts directly with P to form flocs of Fe_3_(PO_4_)_2_, and Al_2_(SO_4_)_3_ assists in the sedimentation of the small-volume flocs. Al_13_ is a significant hydrolysis product of Al^3+^, and Fe and P would substitute for the peripheral Al^VI^ of the Al_13_ structure to form stable Fe–O–Al covalent bonds.

## Introduction

In 2017, 95,400 tons of total phosphorus (TP) was released from domestic sources in China, which was the second source of TP after agricultural sources (released 212,000 tons of TP)^1^. Phosphorus (P) is one of the essential nutrients for plant growth. We have investigated the urban sewage plants around Zhengzhou city, and the average TP content of biological treatment effluent was 0.203 mg/l (Table SI1). However, slow-flowing water could be eutrophic when the P concentration exceeds 0.02 mg/l^2^. The main phenomena of eutrophication in water bodies include algae and plankton blooms and a large number of fish and shrimp deaths due to reduced dissolved oxygen^3,4^. Therefore, advanced removal of P from biotreated sewage effluent is an important method to prevent and control eutrophication in water bodies.

The main methods commonly used to remove P from wastewater are biological treatment, adsorption and chemical precipitation^5^. In the biological P removal treatment, microorganisms accumulate P beyond normal requirements for metabolic processes^6^. However, the TP removal efficiency of the biological treatment is frequently hindered by different operational and system constraints. In reports, the TP removal of biological treatment was susceptible to effects by the temperature, hydraulic retention time, and reaction volume^7–9^. In addition, TP in the effluent of biological treatment tends beyond the permissible limit. Falahati-Marvast and Karimi-Jashni reported that the optimal TP content of 0.7 mg/l in the effluent of a pilot-scale bioreactor exceeded the discharge limit of P for urban wastewater treatment plant (GB18919-2002)^10^. Therefore, physicochemical processes of P removal are usually combined with biological treatment in application practice.

Physicochemical methods such as adsorption and precipitation are common advanced P removal technology. However, the limitation of adsorption is the P sorption capacity of the absorbents^11^. Han et al. reported a decrease in the P removal efficiency of absorbent with a period of usage because of P saturation^12^. Chemical precipitation is one of the most common advanced P removal techniques. The metal salts, such as ferric chloride and alum, combine with P in the wastewater, and the coagulation flocs are removed by sedimentation or filtration. Chemical precipitation has been proven to be an effective process and is widely applied in urban wastewater treatment plants^13^. Li et al. reported that ferric chloride had a high efficiency and stable P removal from wastewater^14^. However, a typical drawback of chemical precipitation is the high cost of using metal salts^15^. In addition, excessive coagulants such as alum will increase the metal concentration in the effluent, which is toxic and harmful to human health^16^. To improve the P removal effect of inorganic coagulants and reduce the dosage of coagulants, a lot of research and application practice has proved that the combination of iron salt and aluminum salt as the composite coagulant is a reliable method of enhanced coagulation^17^.

Composite coagulants are an effective method of advanced P removal. The composite coagulant is a "polymerized, compounded, multi-functional" traditional coagulant. The preparation process of composite coagulant is simple but can enhance the coagulation effect^18,19^. The benefits of composite coagulants include low mud production, wide pH range, and less temperature dependence, which are the hot spots of current research in wastewater treatment. Zhao et al. reported that the composite coagulant prepared by red mud was environmentally and economically viable for advanced P removal in urban wastewater^15^. Composite coagulants include inorganic-inorganic, organic–inorganic, and organic-organic composite coagulants^20,21^. Among them, the inorganic-inorganic composite coagulant has lower cost and easier control of the coagulation process, and its application in practical engineering is feasible. Yang et al. reported that inorganic polymeric coagulants can aid particle surface charge neutralization and sweep flocculation due to the synergistic effects of metal ions to promote P stability^22^. Ma et al. reported the reduction of Al and Fe residual in the treated effluent of Al/Fe-based composite coagulant^23^. Composite coagulants of Fe salts and Al salts can promote the hydrolysis degree of metal ions to enhance the ability of the ionic layer compression, electrical neutralization, adsorption-bridging, and sweep coagulation, thereby improving the P removal effect^24,25^. However, current research focuses on the preparation of polymer composite coagulants^26^. It is interesting to explore the combining process of composite coagulant with P in actual wastewater to help determine the treatment strategy of advanced P removal.

The objective of this study was to compare the effect of dosage, Fe/Al mass ratio, and pH on the TP removal efficiency of Fe–Al composite coagulants (FeCl_3_-AlCl_3_, FeSO_4_-Al_2_(SO_4_)_3_) and single coagulants (FeCl_3_, FeSO_4_, AlCl_3_, and Al_2_(SO_4_)_3_). We investigated the transformation of P forms under the coagulation treatment and calculated the hydrolysis speciation of Fe^3+^, Fe^2+^, and Al^3+^ at a pH range by Visual MINTEQ. The coagulation precipitate was observed using the scanning electron microscope (SEM), Fourier transformed infrared (FTIR), and X-ray photoelectron spectroscopy (XPS) to discuss the process of the composite coagulant combining with P.

## Materials and methods

### Materials and water sample

#### Preparation of coagulants

Ferric chloride (FeCl_3_, Rhawn), ferrous sulphate (FeSO_4_, Rhawn), aluminum chloride (AlCl_3_, Rhawn), and aluminum sulphate (Al_2_(SO_4_)_3_, Rhawn) were dissolved in ultrapure water and freshly prepared with 1.5 g/l coagulant solution, respectively. FeCl_3_-AlCl_3_ and FeSO_4_-Al_2_(SO_4_)_3_ composite coagulants were prepared by mixing FeCl_3_ and AlCl_3_, FeSO_4_ and Al_2_(SO_4_)_3_ solutions at the mass ratios of 0.5, 1, and 2, respectively. The composite coagulant solution was diluted to 1.5 g/l after stirring rapidly for 30 min on a magnetic stirrer (TJ-6, Hengling). Diluted hydrochloric acid (HCl, Luoyang chemical reagent factory) and sodium hydroxide (NaOH, Rhawn) were used to adjust the pH. All chemicals used above were of analytical grade. Ultrapure water was prepared by a water purification apparatus (GWB-2, Persee).

#### Water sample

According to the Ammonium Molybdate Spectrophotometric Method (GB11893-89), the measured water sample should be acidified pretreatment to inhibit the effect of microbial metabolism on TP determination. However, acidification will change the P forms of the water sample^27^. In this study, to investigate the process of combining composite coagulants with different P forms in the actual wastewater, the water sample was obtained from the effluent of a pilot-scale self-cleaning activated bio-filter (Fig. SI1). Table 1 shows the water quality of the effluent when the self-cleaning activated bio-filter was stable. Comparing the P forms composition, effluent from the self-cleaning activated bio-filter could properly represent the urban sewage effluent (Table SI1).

**Table 1:** Water quality of self-cleaning activated bio-filter.

Water sample	TP (mg/l)	Total dissolved P (mg/l)	Dissolved ortho P (mg/l)	Dissolved organic P (mg/l)	Total suspended P (mg/l)	Suspended ortho P (mg/l)	Suspended organic P (mg/l)
Effluent	0.519	0.404	0.39	0.014	0.115	0.003	0.112

### Coagulation experiments

#### Effect of coagulant dose on TP removal efficiency

A six-paddle stirrer (JJ-3A, Olabo) was used in the coagulation experiments. In each jar test, 300 ml of self-cleaning activated bio-filter effluent was added with 5, 10, 15, 20, 25, and 30 mg/l of composite coagulant solutions with Fe/Al mass ratios of 0.5, 1, and 2, respectively. The mixtures were rapidly stirred for 1 min at 200 rpm, followed by a slow stirring for 30 min at 30 rpm, then settling for 30 min at room temperature (25 ± 1 ℃). After settling, the supernatant samples were obtained to measure TP concentration and P forms.

We did not additionally add humic substances to the effluent of the self-cleaning activated bio-filter because humic substances had little effect on P removal by coagulants (Fig. SI2).

#### Effect of pH on TP removal efficiency

In each jar test, a pH meter (PHS-3C, Lei-ci) measured the pH of self-cleaning activated bio-filter effluent. Solutions of HCl and NaOH were used to adjust the pH of the raw water samples to the target pH (4–9). 15 mg/l composite coagulants with Fe/Al mass ratios of 0.5, 1, and 2 were added to the pH-adjusted self-cleaning activated bio-filter effluent, respectively.

### Analytical methods

#### Analysis of phosphorus forms

**Figure 1:**
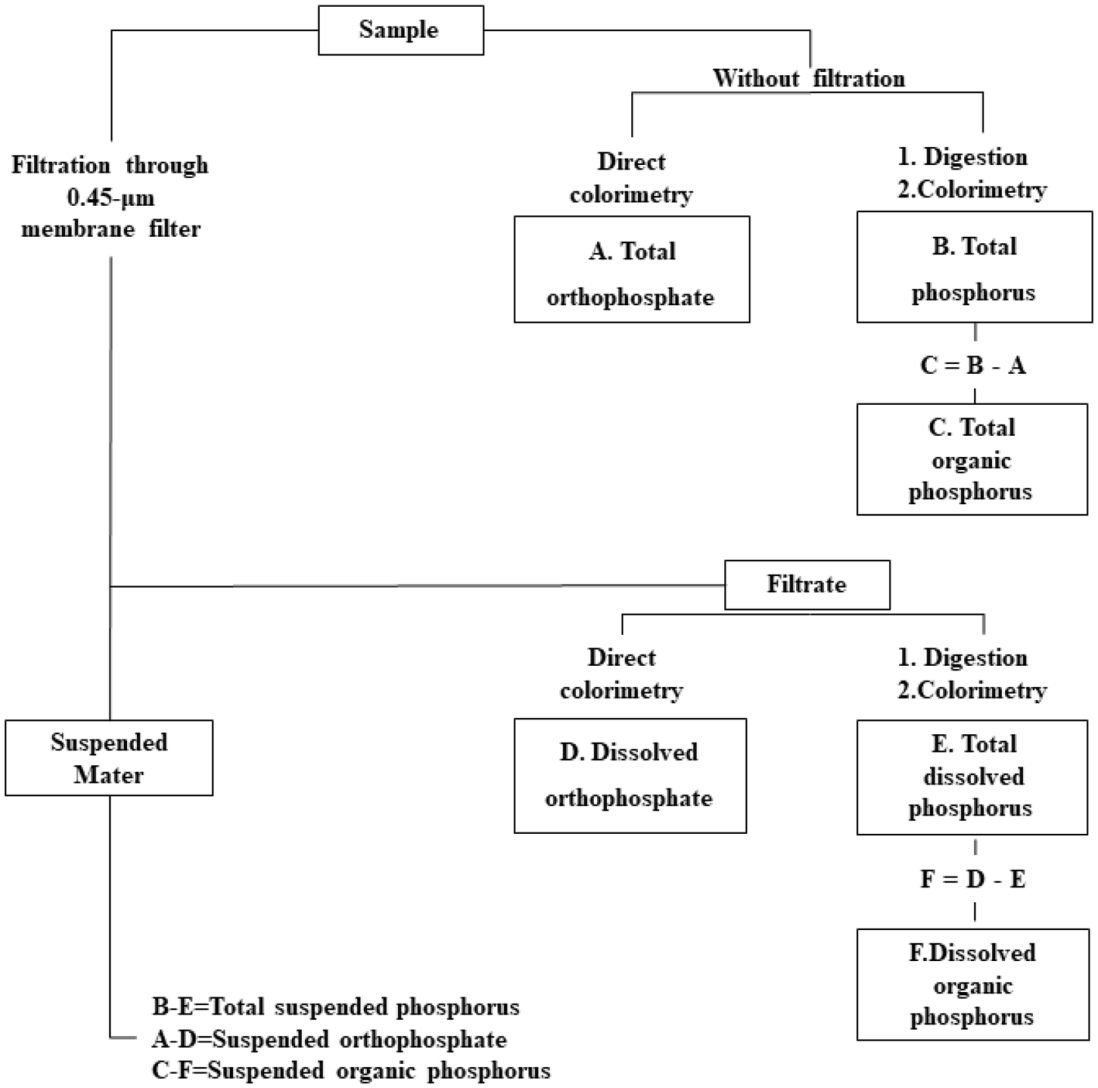
Steps of analysis of phosphate forms.

The analysis of P forms is divided into two steps: converting the P forms of interest to dissolved orthophosphate and determining the concentration of dissolved orthophosphate^28^. The collected water sample of the coagulation experiment was digested at 120 ℃ for 30 min, and then the TP was measured by colorimetry with a UV–vis spectrophotometer (UV-6300, Mapada). Total dissolved P was determined by colorimetry through a 0.45 μm filter membrane before digestion. Total suspended P was calculated as the difference between the TP and total dissolved P. The total orthophosphate was measured by direct colorimetry, and the total dissolved orthophosphate was measured by colorimetry through a 0.45 μm filter membrane without digestion. The total organic P was calculated as the difference between the TP and orthophosphate^29^. The analysis process of the P forms is shown in Fig. 1.

#### Analysis of hydrolysis speciation

Visual MINTEQ 3.1 was used to calculate the distribution coefficients (δ) of Me^3+^, Me(OH)^2+^, Me(OH)_2_^+^, Me(OH)_3_, and the polynuclear hydroxyl complexes of Al^3+^, Fe^3+^ and Fe^2+^ at a pH range. In this study, the setup conditions of the software were pH range from 1 to 13, temperature 25 ℃, ionic strength 0.001, and initial metal ions concentration 1 mol^30^.

#### Analysis of coagulation precipitate

After settling, the coagulation precipitates were carefully taken from the beaker and dried for several hours. The morphology and structure of the precipitates were observed with a scanning electron microscope (Apreo 2, Thermo Fisher). The characteristics of the precipitates were analyzed by an FTIR spectrometer (Nicolet iS50, Thermo Fisher). The chemical compositions of the precipitates were analyzed by an X-ray photoelectron spectrometer (K-Alpha, Thermo Fisher).

## Results and discussion

### Effect of coagulant dosage

**Figure 2:**
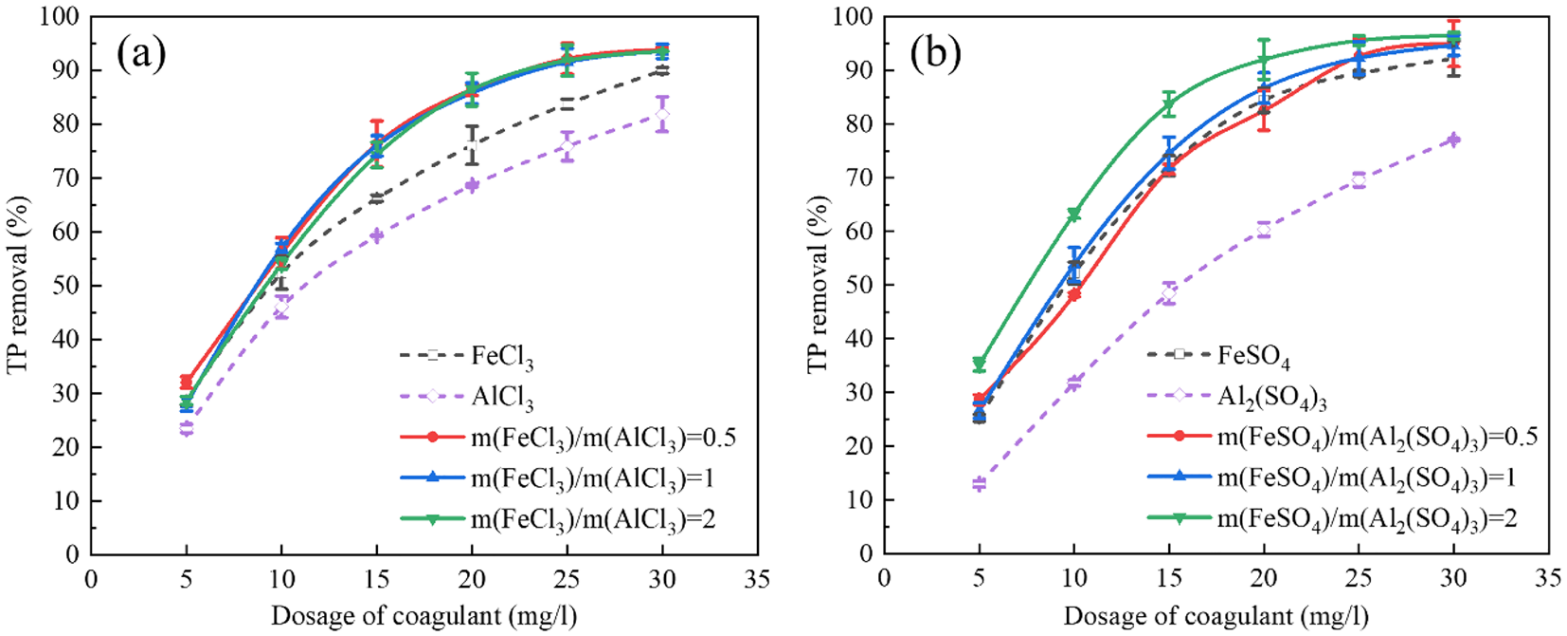
TP removal rate of FeCl_3_-AlCl_3_ (**a**), FeSO_4_-Al_2_(SO_4_)_3_ (**b**) composite coagulants.

In advanced P removal, coagulant dosage is essential in affecting TP removal efficiency, application cost, and toxicity in the effluent. Figure 2a illustrates that the TP removal rate of the FeCl_3_-AlCl_3_ composite coagulant is higher than FeCl_3_ and AlCl_3_ only at 10–30 mg/l. Compared with single coagulants of FeCl_3_ and AlCl_3_, the FeCl_3_-AlCl_3_ composite coagulant can significantly reduce the dosage and cost of P removal by chemical precipitation. For the 90% TP removal, the optimal dosages of single FeCl_3_, AlCl_3_, and FeCl_3_-AlCl_3_ are 30.01, 38.52, and 21.85 mg/l, respectively. The TP removal rate of AlCl_3_ is lower than FeCl_3_. As a result, Fe^3+^ have a higher affinity for P and hydrolyses more rapidly than Al^3+^^31^. At the same time, the TP removal rates of the composite coagulant at the dosage of 5 mg/l and 30 mg/l (32.08%, 93.89%) are approaching the FeCl_3_ only (28.64%, 89.99%), respectively. Yang et al. reported that the excess hybrid coagulant had a less beneficial effect on turbidity removal^32^. In reports, coexisting anions had a limited influence on P removal by physicochemical methods^33^. However, the TP removal rates of the FeCl_3_-Al_2_(SO_4_)_3_ and the FeCl_3_-PAC are less than the FeCl_3_-AlCl_3_ (Fig. SI3). This phenomenon may be attributed to the reduced solubility of the precipitate due to the common ion effect.

Figure 2b shows that the TP removal rate of the FeSO_4_-Al_2_(SO_4_)_3_ composite coagulant at the FeSO_4_/Al_2_(SO_4_)_3_ mass ratio of 2 is significantly higher than single FeSO_4_ and Al_2_(SO_4_)_3_. The TP removal rate of FeSO_4_-Al_2_(SO_4_)_3_ composite coagulant is 83.71% higher than FeSO_4_ (72.2%) and Al_2_(SO_4_)_3_ (48.48%) at the dosage of 15 mg/l. With reducing the FeSO_4_/Al_2_(SO_4_)_3_ mass ratio, the TP removal rate of the FeSO_4_-Al_2_(SO_4_)_3_ composite coagulant shows a decreasing tendency. The Fe/Al mass ratio significantly impacts the P removal efficiency of Fe^2+^-based composite coagulants. For the 90% TP removal, the optimal dosages of single FeSO_4_, Al_2_(SO_4_)_3_, and FeSO_4_-Al_2_(SO_4_)_3_ are 25.43, 41.05, and 18.25 mg/l, respectively. Guan et al. reported that the application of Fe^2+^ with metal ions could increase the surface charge and produce more precipitated ferrous hydroxide or ferric hydroxide^34^. Compared with FeSO_4_-Al_2_(SO_4_)_3_, the TP removal rates of the FeSO_4_-AlCl_3_ and FeSO_4_-PAC are less improved than the single FeSO_4_ (Fig. SI4).

We have investigated the relationship between injection orders of Fe-Al composite coagulants and P removal (Fig. SI5). There is little influence of injection order on P removal efficiency, so we will not discuss the injection order in the following. We have also tested the P removal efficiencies of PFS-AlCl_3_ and PFS-Al_2_(SO_4_)_3_, and the test results show that FeCl_3_-AlCl_3_ and FeSO_4_-Al_2_(SO_4_)_3_ have the most obvious promotion effect on P removal (Fig. SI6).

### Variations of phosphorus forms

**Figure 3:**
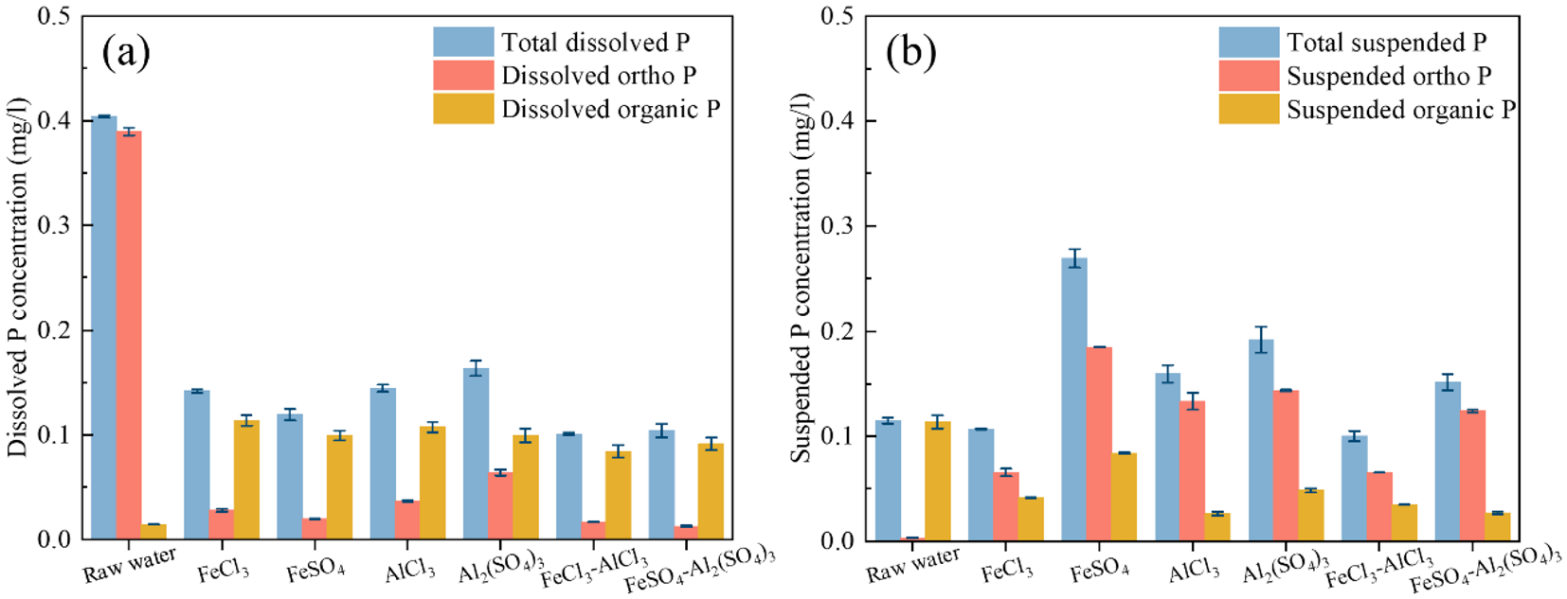
Effect of coagulants on the phosphorus forms in biological effluent.

Investigating the change of P forms under coagulation treatment contributes to exploring the combining process of coagulant with P. Figure 3a and b illustrate that the dissolved orthophosphate and suspended organic P are the primary P forms in the self-cleaning activated bio-filter effluent. Metal salts as coagulants are added to the wastewater to form crystalline precipitates, which adsorb the dissolved P on the surface of the precipitates and transform to suspended P^35^. The suspended P is subsequently separated by gravity. The dissolved orthophosphate is mostly removed after dosing 15 mg/l coagulant, and the FeSO_4_-Al_2_(SO_4_)_3_ composite coagulant achieved the highest dissolved orthophosphate removal rate of 96.76%. An increase of suspended orthophosphate in the coagulation effluent indicates that the coagulant combines with dissolved orthophosphate to form suspended orthophosphate flocs^36^. There are 76.79% and 57.14% removal of suspended organic P under AlCl_3_ and Al_2_(SO_4_)_3_ treatment, respectively. The removal of suspended organic P is mainly by adsorption-bridging and sweep coagulation^37^. Compared with FeSO_4_ of 25% removal for suspended organic P, it indicates the removal of suspended P by FeSO_4_ through ionic layer compression and electrical neutralization. The suspended orthophosphate content of the FeSO_4_ coagulation effluent is 0.185 mg/l, and the FeSO_4_–Al_2_(SO_4_)_3_ composite coagulant reduces the suspended orthophosphate content by 32.8% compared to FeSO_4_ only. It indicates that the Fe^2+^ combines with the P to form the slight and hard settling flocs of Fe_3_(PO_4_)_2_ in the FeSO_4_ removal P process, and the Al_2_(SO_4_)_3_ will promote the settling of Fe_3_(PO_4_)_2_ flocs. In this study, we observed the increased dissolved organic P concentration of the coagulation effluent, with a 6.6 times growth rate of dissolved organic P by FeCl_3_. This phenomenon is due to the limitation of the P forms analytical method, which ignores that a proportion of inorganic P, such as polyphosphates, cannot be measured by direct colorimetry^28^.

### Effect of pH

**Figure 4:**
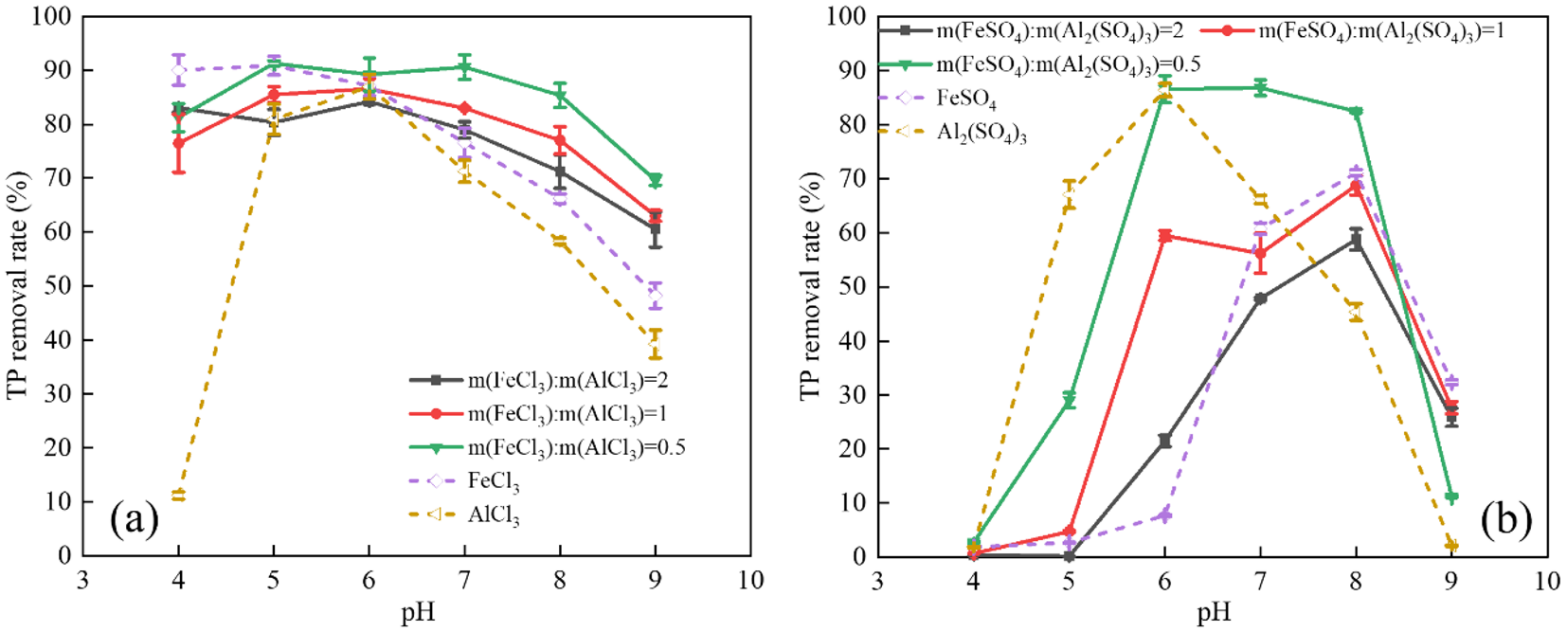
Effect of pH on the TP removal rate of FeCl_3_-AlCl_3_ (**a**), FeSO_4_-Al_2_(SO_4_)_3_ (**b**) composite coagulants.

pH depends on the degree of reaction between hydroxyl and metal ions, thus affecting the bridging flocculation^38^. Figure 4a and b show that the TP removal rates of FeCl_3_-AlCl_3_ and FeSO_4_-Al_2_(SO_4_)_3_ composite coagulants are higher than single coagulants (FeCl_3_, FeSO_4_, AlCl_3_, and Al_2_(SO_4_)_3_) when 6 < pH < 9. Li et al. reported that composite coagulants would hydrolyze to produce long, complex, and stable reaction bonds, which are difficult to destroy by the change in pH^39^. The optimal TP removal of FeCl_3_-AlCl_3_ and FeSO_4_-Al_2_(SO_4_)_3_ are 91.31% and 86.82% at pH 5 and 7, respectively. In a weakly acidic or neutral solution, the adsorption sites on the surface of the hydroxide produced by the hydrolysis of metal ions could adsorb a large amount of P. However, the production of metal hydroxides from coagulants would be inhibited in acidic or alkaline solutions. The composite coagulants show a wider pH range with better P removal efficiency. The Fe/Al mass ratio is negatively correlated with the TP removal rate at pH 4–9. The TP removal rates of FeSO_4_-Al_2_(SO_4_)_3_ are 86.55% and 21.5%, corresponding to the FeSO_4_/Al_2_(SO_4_)_3_ mass ratio of 2 and 0.5, respectively. FeSO_4_ is prone to change in pH because Fe^2+^ reacts with OH^-^ in the solution to form soluble Fe(OH)_2_, which is easily oxidized to Fe(OH)_3_ by dissolved oxygen in wastewater. The oxidation reaction is inhibited at low pH due to insufficient OH^-^ in the wastewater, resulting in the decreased removal of TP^40^. pH affects the hydrolysis product of metal ions and P form in solutions^41^. According to the dissociation constants of phosphate (K(H_3_PO_4_) = 2.15; K(H_2_PO_4_^–^) = 7.2; K(HPO_4_^2–^) = 12.38), at 2.15 < pH < 7.2, the predominant species of P in the solution is H_2_PO_4_^–^ (Fig. SI7), which facilitates combining to metal hydroxide with the positively charged ^42^.

**Figure 5:**
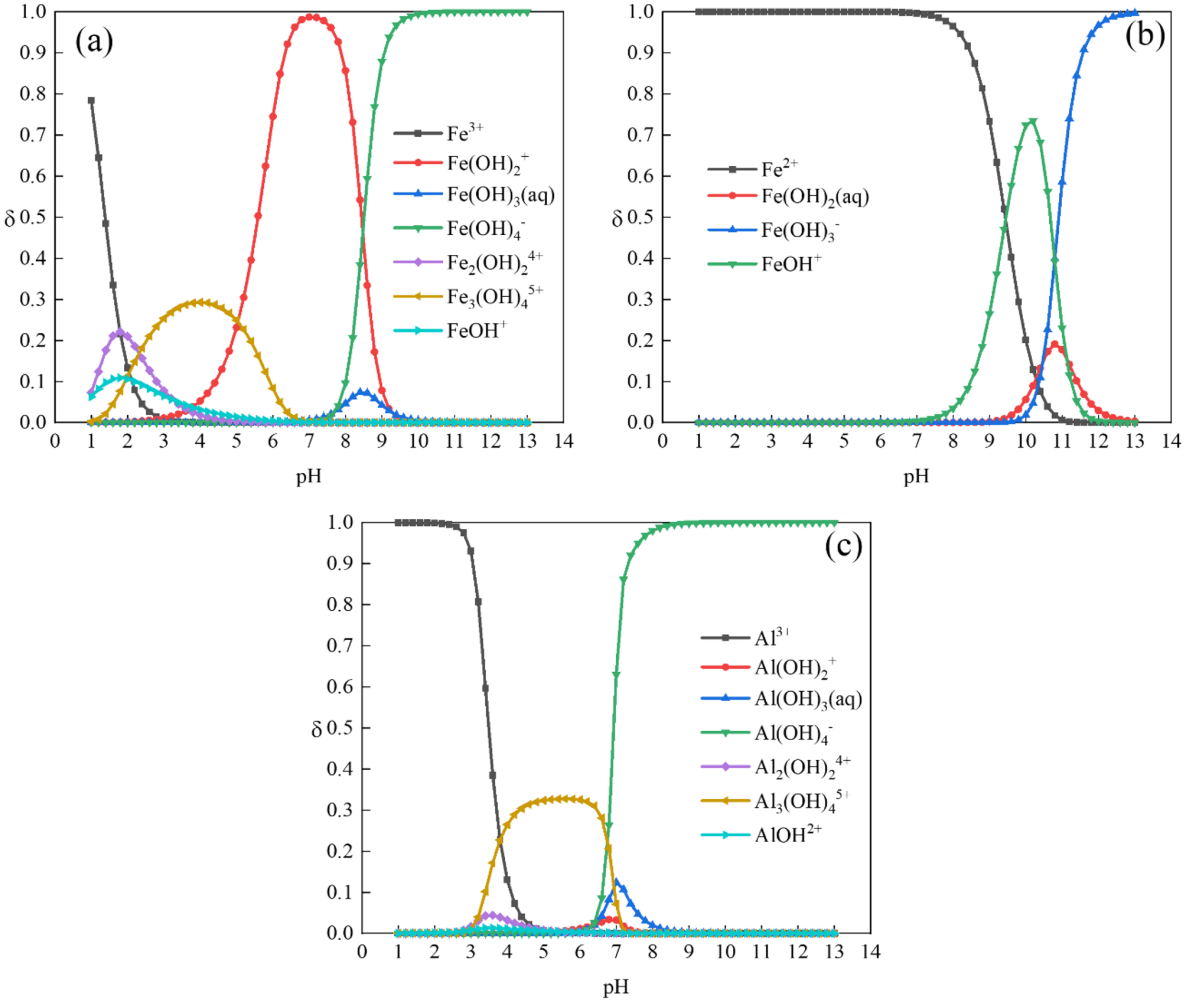
Hydrolysis speciation of Fe^3+^ (**a**), Fe^2+^ (**b**) and Al^3+^ (**c**) with pH.

Figure 5a shows that the hydrolysis products of Fe^3+^ are mainly mononuclear hydroxides such as Fe(OH)_2_^+^, Fe(OH)_4_^–^ or polynuclear hydroxyl complexes such as Fe_3_(OH)_4_^5^^+^. Figure 5b shows that the hydrolysis products of Fe^2+^ are mainly Fe^2+^ and mononuclear hydroxides such as FeOH^+^ and Fe(OH)_3_^–^. Fe^2+^ has lower P removal efficiency than Fe^3+^ in practical applications because Fe^2+^ does not hydrolyze to produce polynuclear hydroxyl complexes^43^. Figure 5c shows that the main hydrolysis products of Al^3+^ are Al(OH)_4_^–^ and Al_3_(OH)_4_^5^^+^, which are similar to the hydrolysis products of Fe^3+^. Compared to Fig. 4a and b, the optimal TP removal rates of Al salts are achieved at the pH range of 5–6, corresponding to the main hydrolysis product of Al^3+^ is Al_3_(OH)_4_^5^^+^. It indicates that the hydroxides produced by Fe^3+^ and Al^3+^ play a significant role in P adsorption, and Fe^2+^ combines directly with P to form the precipitate of Fe_3_(PO_4_)_2_.

### SEM analysis

**Figure 6:**
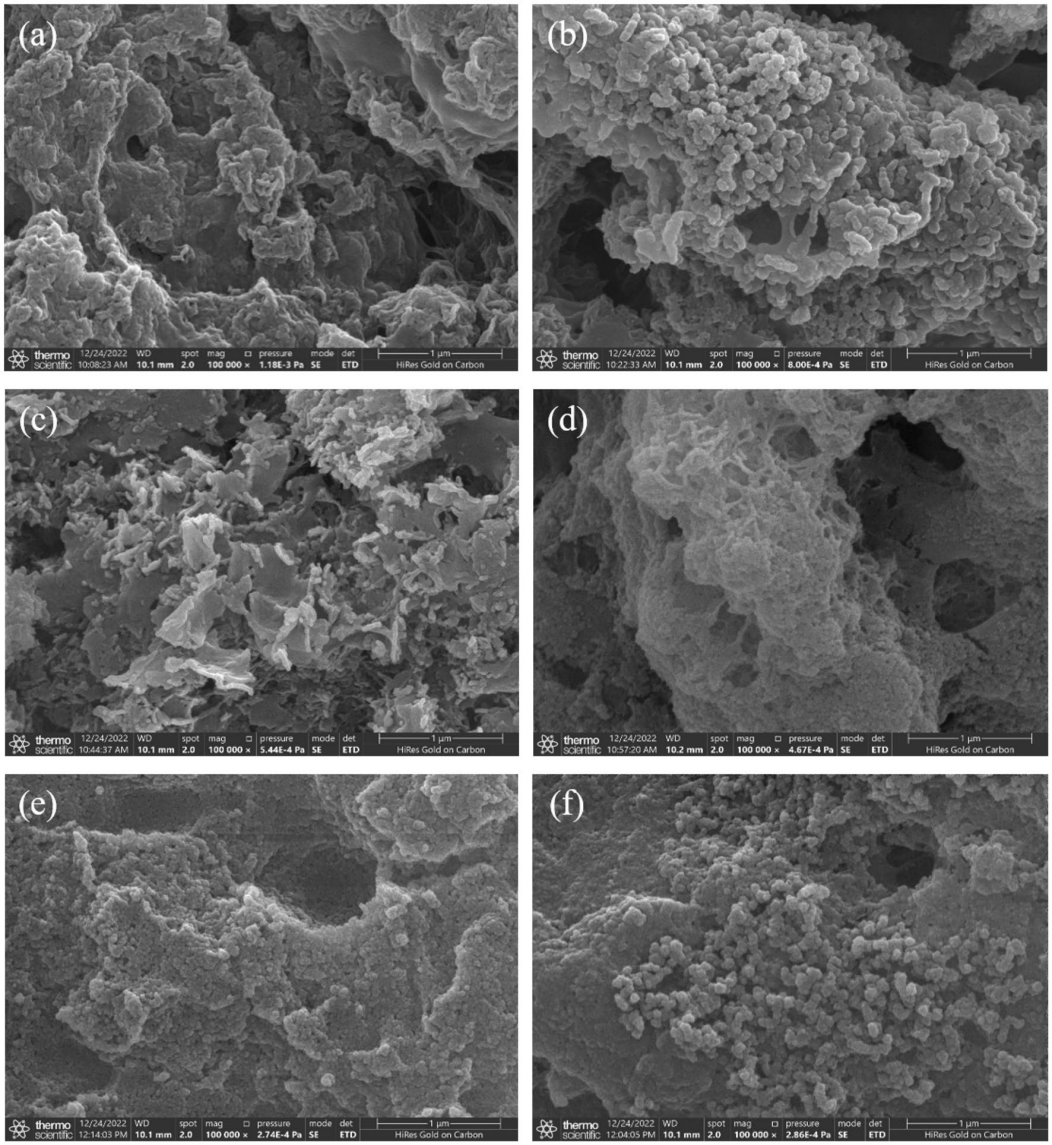
SEM analysis of the precipitate collected in single coagulants (FeCl_3_ (**a**), FeSO_4_ (**b**), AlCl_3_ (**c**), Al_2_(SO_4_)_3_ (**d**)) and composite coagulants (FeCl_3_-AlCl_3_ (**e**), FeSO_4_-Al_2_(SO_4_)_3_ (**f**)).

Figure 6a shows that the structure of the FeCl_3_ coagulation precipitate is porous and loose with an irregular and partly smooth surface. Moreover, the FeSO_4_ coagulation precipitate consists of Fe_3_(PO_4_)_2_ particles with a diameter of around 0.1 μm (Fig. 6b). Figure 6c shows that the morphology of the AlCl_3_ is petal-like with an average length of 0.25 μm, similar to threadlike NaCl crystal^44^. The distinctions in the structure of the AlCl_3_ and Al_2_(SO_4_)_3_ (Fig. 6d) precipitates are due to the different anions, such as Cl^-^ and SO_4_^2^^–^, involved in combining the coagulant with the contaminant^45^. Figure 6e shows that the structure of the FeCl_3_-AlCl_3_ coagulation precipitates is significantly different from the FeCl_3_ and AlCl_3_. The change in structure indicates that the composite coagulant modifies the binding mode of P. Figure 6f shows that the structure of the FeSO_4_ precipitate is similar to the precipitate of FeSO_4_-Al_2_(SO_4_)_3_, which consists of several 0.1 μm diameter Fe_3_(PO_4_)_2_ particles. According to Sect. "Variations of phosphorus forms", it is reasonable to speculate that the P removal process of FeSO_4_-Al_2_(SO_4_)_3_ composite coagulant is mainly the reaction of Fe^2+^ with P to form Fe_3_(PO_4_)_2_ flocs, and the hydrolysis product of the Al_2_(SO_4_)_3_ performs the adsorption-bridging and sweep coagulation to promote the Fe_3_(PO_4_)_2_ flocs settling.

### FTIR analysis

**Figure 7:**
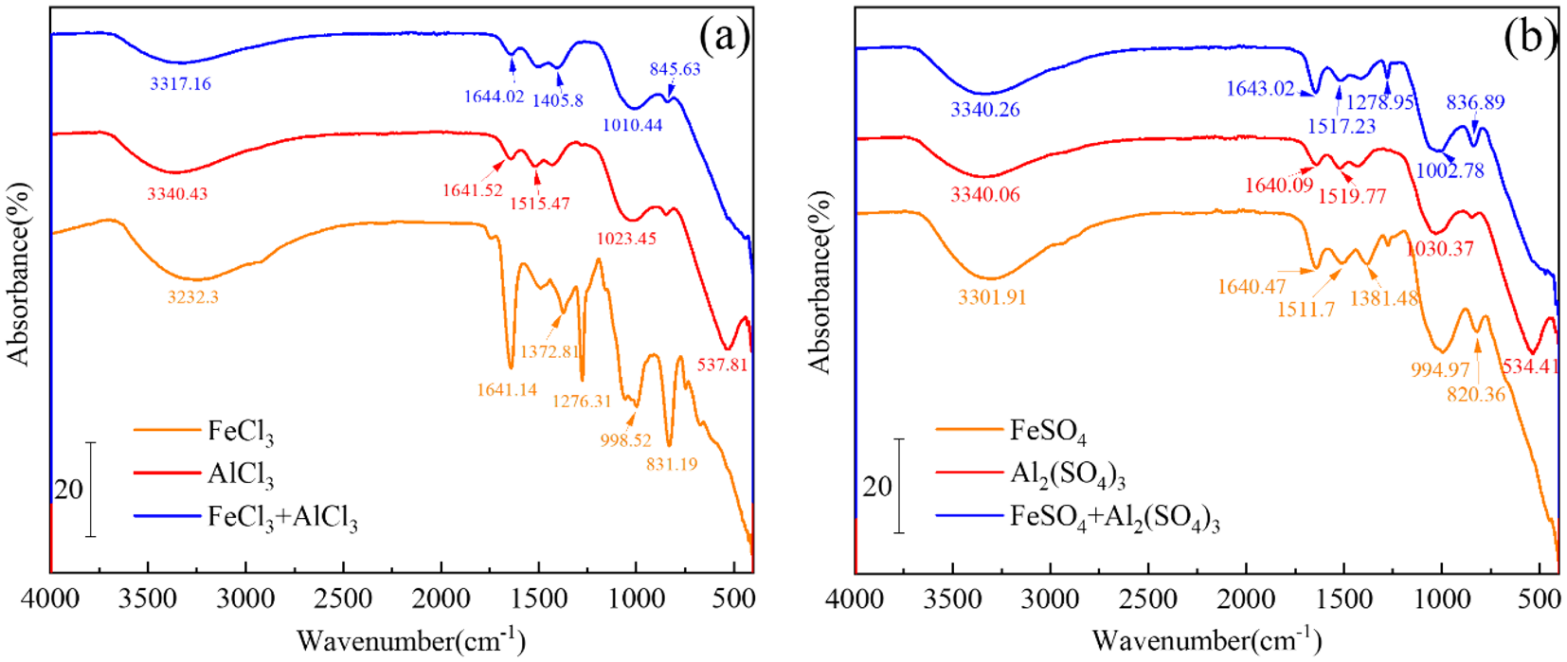
FTIR spectra of the precipitates collected in FeCl_3_-AlCl_3_ (**a**), FeSO_4_-Al_2_(SO_4_)_3_ (**b**) composite coagulants.

As shown in Fig. 7, the stretching vibration at 3300 cm^–1^ is assigned to the O–H, which is due to the absorbed water and hydroxyl group on the surface of the metal ions hydrolysis product or the adsorbed substance. The strong bending vibration at 1600 cm^–1^ is assigned to physically adsorbed H_2_O^14^. The strong bending vibrations at 1510 to 1210 cm^–1^ are assigned to the NO_3_, which is due to the absorption of NO_3_^–^ from the nitrogen source of the simulated wastewater and biological metabolism. The stretching vibration at 1000 cm^–1^ is assigned to the P–OH due to the phosphorus absorption by the polynuclear hydroxyl complexes of the metal ions hydrolysis produced. For the Fe^3+^/Fe^2+^ salts and the Fe-Al composite coagulants coagulation precipitates, the stretching vibration at 830 cm^–1^ is assigned to the P–O. It indicates that Fe–O–P is formed due to the direct reaction of the Fe^3+^/Fe^2+^ with PO_4_^3–^. And for the Al^3+^ salt coagulation precipitate, the absorption band at 535 cm^–1^ is assigned to the Al–O vibrations of aluminum in the octahedral coordination^46^.

### XPS analysis

**Figure 8:**
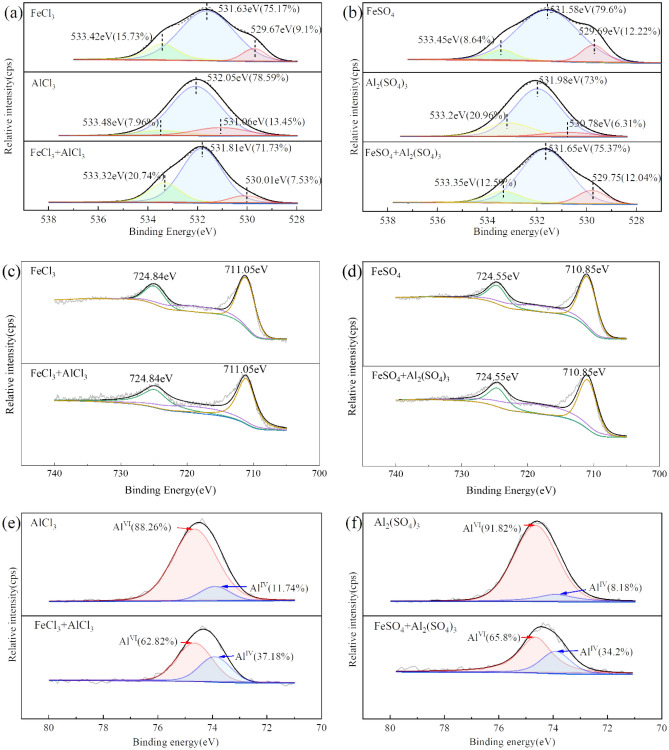
O1s spectra (**a**,**b**), Fe2p spectra (**c**,**d**), and Al2p spectra (**e**,**f**) of the precipitate collected in FeCl_3_-AlCl_3_, FeSO_4_-Al_2_(SO_4_)_3_ composite coagulants.

C, O, Fe, Al and P are the primary constituent elements of coagulation precipitate (Fig. SI8). As shown in Fig. 8a and b, the O1s spectra of the precipitate are deconvoluted into three peaks by the XPSPEAK41. The red peak at the bonding energy of 530 eV is assigned to O in Fe–O–Fe and Al–O–Al, which is due to Fe^3+^, Fe^2+^, and Al^3+^ directly binding with O. The blue peak at the bonding energy of 532 eV is assigned to O in Fe–O–H and Al–O–H, which is due to Fe and Al binding with O in the hydroxyl group ^47^. The yellow peak at the bonding energy of 533.3 eV is assigned to O in H_2_O due to the adsorption of the hydroxyl complexes. The relative area of the peak represents the content of the elemental form in the precipitate, demonstrating that the primary components of the coagulation precipitate are Fe(OH)_3_, Al(OH)_3_, and polynuclear hydroxides^48^. The peak of P2p spectra of the coagulation precipitate is located at 133.4 eV, which is located between the peaks of the Fe salts precipitate (at 133.14 eV) and the Al salts precipitate (at 133.75 eV) (Fig. SI9).

Figure 8c and d show the Fe2p spectra of the coagulation precipitate. It indicates that the peaks at the bonding energies of ~ 711 eV and ~ 724.5 eV are assigned to the Fe2p_3/2_ and Fe2p_1/2_, respectively. The positions of the Fe2p_3/2_ and Fe2p_1/2_ peaks are determined by the element valence state of the Fe^49,50^. The energy separation between Fe2p3/2 and Fe2p_1/2_ is 13.5 eV, which is in agreement with FePO_4_ in the report^51^. After the deconvolution, the peaks at the bonding energy of 711.05 eV and 724.84 eV are assigned to FeOOH, and the peak at the bonding energy of 718.55 eV is assigned to Fe(OH)_3_^52^.

**Figure 9:**
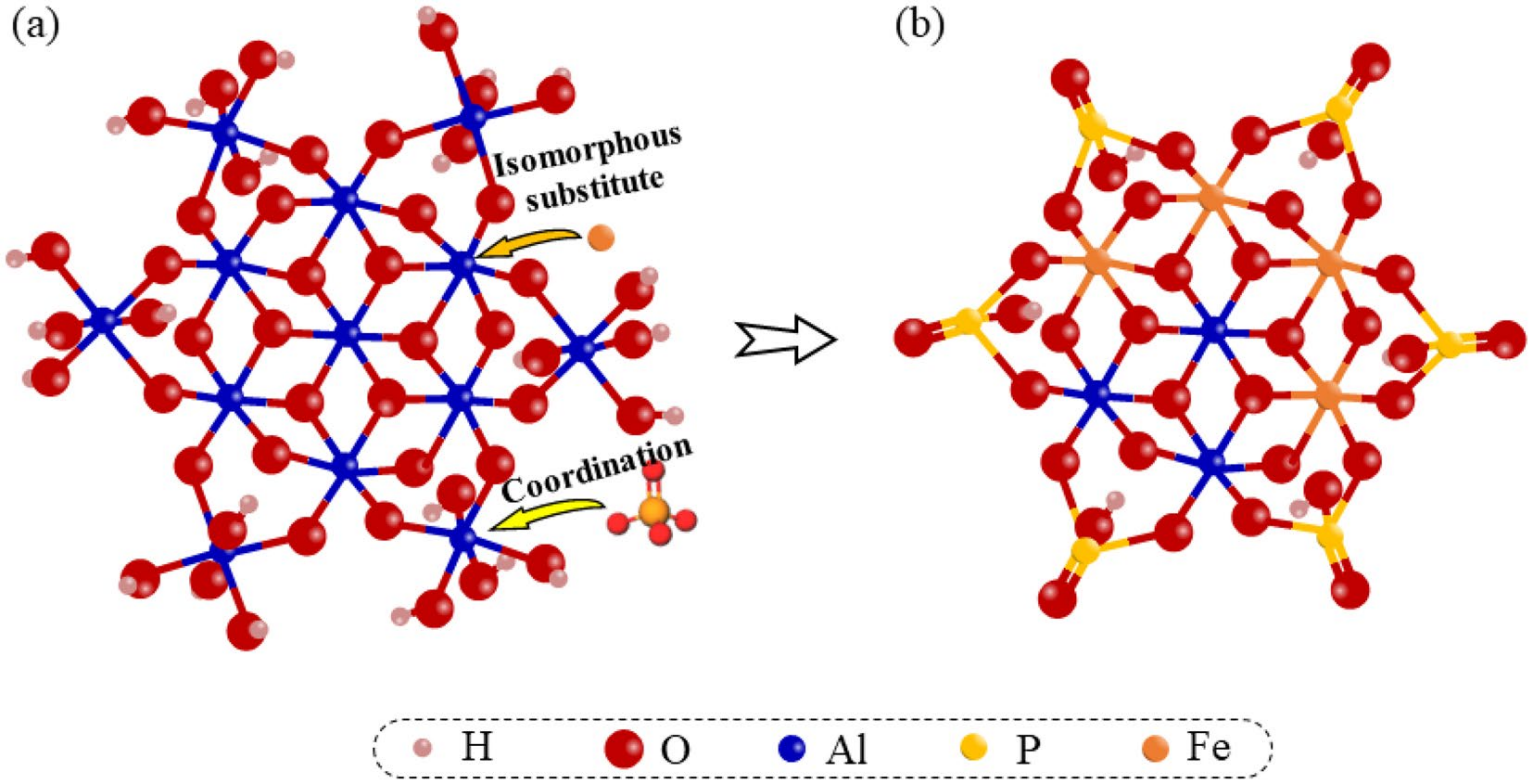
The flat structure of Al_13_ (**a**) and Al_13_ is substituted with Fe and phosphorus (**b**).

Based on Fig. 8e and f, the Al2p spectra of the coagulation precipitate are deconvoluted into two peaks at the bonding energies of 73.89 eV and 74.64 eV, which are assigned to tetrahedrally coordinated Al (Al^IV^) and octahedrally coordinated Al (Al^VI^), respectively. The Al^IV^ has a lower bonding energy than the Al^VI^^53^. During the hydrolysis process of Al^3+^, the metastable [AlO_4_Al_12_(OH)_24_(H_2_O)_12_]^7+^ (Al_13_) is a significant intermediate, with the structure of a central Al^IV^ is surrounded by 12 peripheral Al^VI^^54^. Therefore, the Al^VI^/Al^IV^ ratio of the Al(OH)_3_ is 12 theoretically. However, in this study, the Al^VI^/Al^IV^ ratio of the coagulation precipitate formed by AlCl_3_ and Al_2_(SO_4_)_3_ are 7.52 and 11.22, respectively. It indicates less content of voluminous Al_13_, and Al(OH)_3_ is the major Al species in the precipitate. In addition, the presence of P may impede the formation of Al^VI^^55^. Compared to the coagulation precipitate formed by the single AlCl_3_ and Al_2_(SO_4_)_3_, the Al^VI^ content decreased by 25.44% and 26.02% in the precipitate of FeCl_3_-AlCl_3_ and FeSO_4_-Al_2_(SO_4_)_3_ composite coagulants, respectively. However, the content of the Al^IV^ remains constant. It indicates that the Fe isomorphous substitutes for the peripheral Al^VI^ and is involved in the coordination process of the Al_13_ with the P. The substitution process is illustrated in Fig. 9.

## Conclusion

In this study, we surveyed the effect of Fe-Al composite coagulants on the removal of different P forms and discussed the mechanism of Fe-Al composite coagulants to enhance the removal rate of TP. The main conclusions are as follows:

1. Compared with single coagulants, the TP removal rate of Fe-Al composite coagulants significantly improved. The coagulant combines with dissolved orthophosphate to form suspended orthophosphate and sedimentation.
2. Fe-Al composite coagulants have a higher optimal TP removal rate than single coagulants when 6 < pH < 9. Polynuclear hydroxyl complexes are the primary hydrolysis product of Fe and Al salts coagulants at pH 6. The adsorption-bridging effect of the metal hydroxides hydrolyzed by Fe^3+^ and Al^3+^ plays a significant role in P removal.
3. FeSO_4_ reacts readily with P to form non-settling Fe_3_(PO_4_)_2_ flocs, and Al_2_(SO_4_)_3_ can promote the sedimentation of the small-volume flocs in FeSO_4_-Al_2_(SO_4_)_3_ composite coagulant. Fe isomorphous substitutes for the peripheral Al^VI^ and is involved in the coordination process of the Al_13_ with the P.

In conclusion, Fe-Al composite coagulants are efficient and feasible processes to remove low P concentrations in urban sewage.

### Supplementary Information

Supplementary Information.

## Data Availability

The datasets used and/or analyzed during the current discussion are available and from the corresponding author upon reasonable request.
