# Supplementary material for: Advanced removal of phosphorus from urban sewage using chemical precipitation by Fe-Al composite coagulants
Source: Sci Rep. 2024 Feb 28;14:4918. doi: 10.1038/s41598-024-55713-2 (PMC10901887; doi:10.1038/s41598-024-55713-2)
Supplement: Supplementary file 1 — Supplementary Information. [file 41598_2024_55713_MOESM1_ESM.docx]

**Supplementary Information**

**Advanced removal of phosphorus from urban sewage using chemical precipitation by Fe-Al composite coagulants**

Hongbin Xu^1^, Songyu Wei^2^, Guoqiang Li^1, *^, Baolei Guo^1^

1. School of Ecology and Environment, Zhengzhou University, Zhengzhou 450001, China;

2. School of Water Conservancy and Transportation, Zhengzhou University, Zhengzhou 450001, China;

**Email address:**

Hongbin Xu: xuhongbin_gy@zzu.edu.cn

Songyu Wei: [871410183@qq.com](mailto:871410183@qq.com)

Guoqiang Li: [liguoqiang@zzu.edu.cn](mailto:liguoqiang@zzu.edu.cn)

Baolei Guo: [w13346882267@163.com](mailto:w13346882267@163.com)

***Corresponding author**:

Guoqiang Li

E-mail: [liguoqiang@zzu.edu.cn](mailto:liguoqiang@zzu.edu.cn)

Mailing address: School of Ecology and Environment, Zhengzhou University, Zhengzhou 450001, China.

**Table SI1** Phosphorus forms in the biological effluent of Zhengzhou WWTPs

| Parameter | WWTP A | WWTP B | WWTP C | WWTP D |
| --- | --- | --- | --- | --- |
| COD (mg/l) | 15 | 14 | 7 | 46 |
| Total nitrogen (mg/l) | 9.95 | 16.51 | 11.13 | 4.17 |
| Total phosphorus (mg/l) | 0.215 | 0.134 | 0.193 | 0.27 |
| Total dissolved P (mg/l) | 0.16 | 0.039 | 0.143 | 0.184 |
| Dissolved ortho P (mg/l) | 0.151 | 0.039 | 0.136 | 0.137 |
| Dissolved organic P (mg/l) | 0.009 | 0 | 0.007 | 0.047 |
| Total suspended P (mg/l) | 0.055 | 0.095 | 0.05 | 0.086 |
| Suspended ortho P (mg/l) | 0.005 | 0.056 | 0.019 | 0.032 |
| Suspended organic P (mg/l) | 0.05 | 0.039 | 0.031 | 0.054 |

**Table SI2** Reagents for artificial simulated wastewater

| Number | Reagent Name | Fineness | Company |
| --- | --- | --- | --- |
| 1 | C_6_H_12_O_6_ | AR | Kemiou |
| 2 | NH_4_Cl | AR | Kemiou |
| 3 | KH_2_PO_4_ | AR | Kemiou |
| 4 | NaNO_3_ | AR | Kemiou |
| 5 | CH_3_COONa | AR | Kemiou |
| 6 | CH_3_OH | AR | Kemiou |
| 7 | H_2_BO_3_ | AR | Kemiou |
| 8 | MnCl_2_∙H_2_O | AR | Kemiou |
| 9 | ZnSO_4_∙7H_2_O | AR | Kemiou |
| 10 | Na_2_SO_4_∙2H_2_O | AR | Kemiou |
| 11 | CuSO_4_∙5H_2_O | AR | Kemiou |
| 12 | CoCl_2_∙6H_2_O | AR | Kemiou |


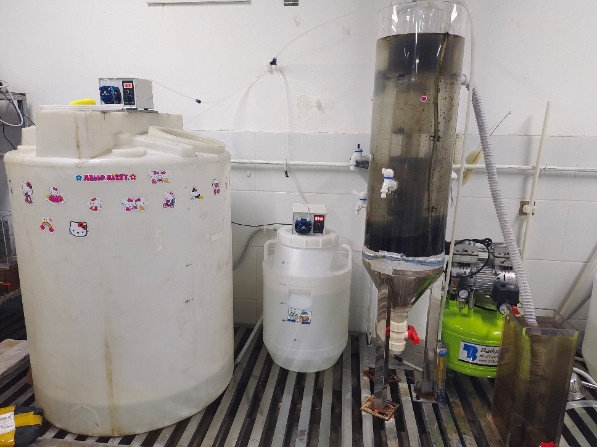


**Fig.SI1** Schematic of Self-cleaning activated bio-filter

**
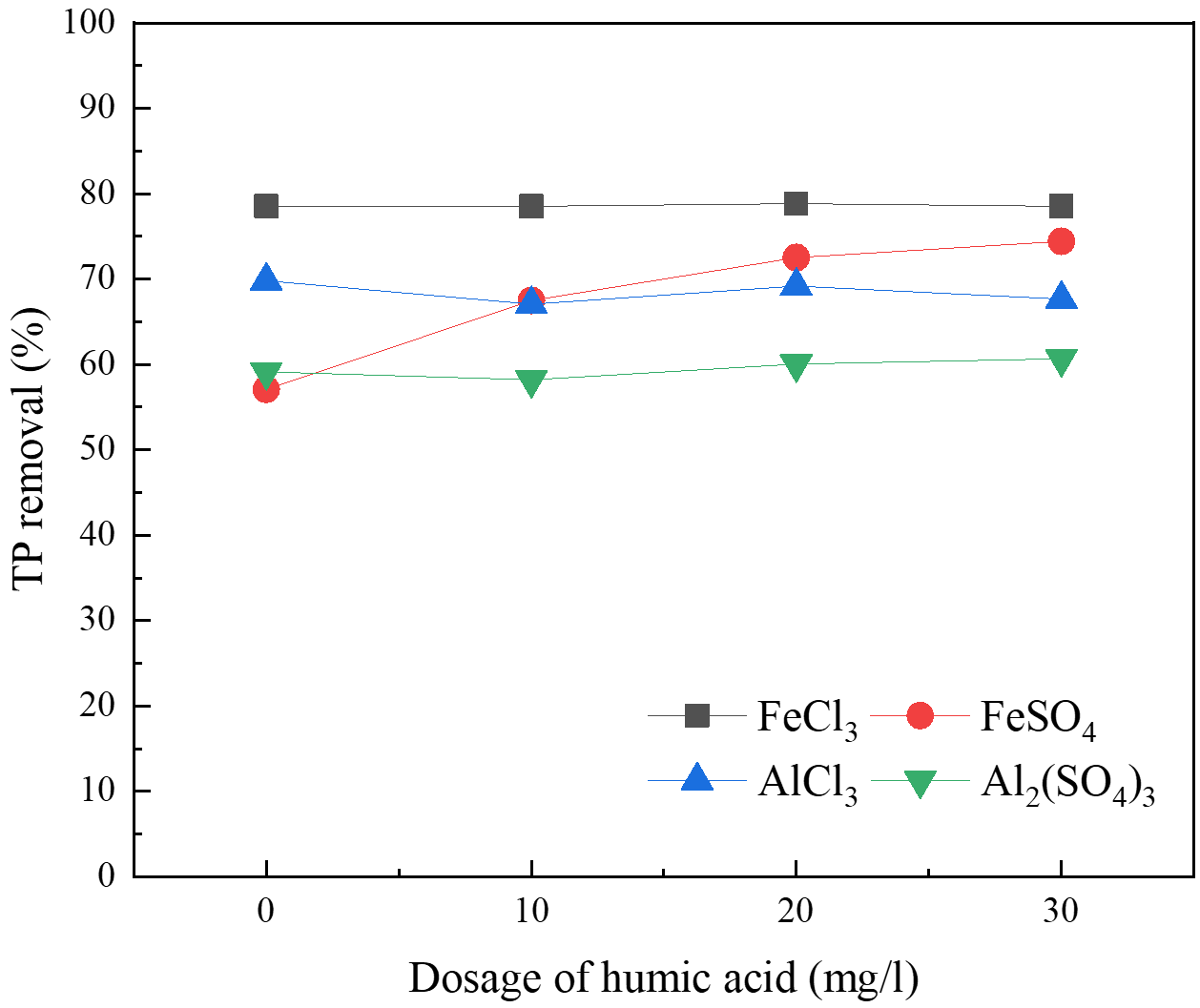
**

**Fig.SI2** Effect of humic acid on the TP removal rate of coagulants


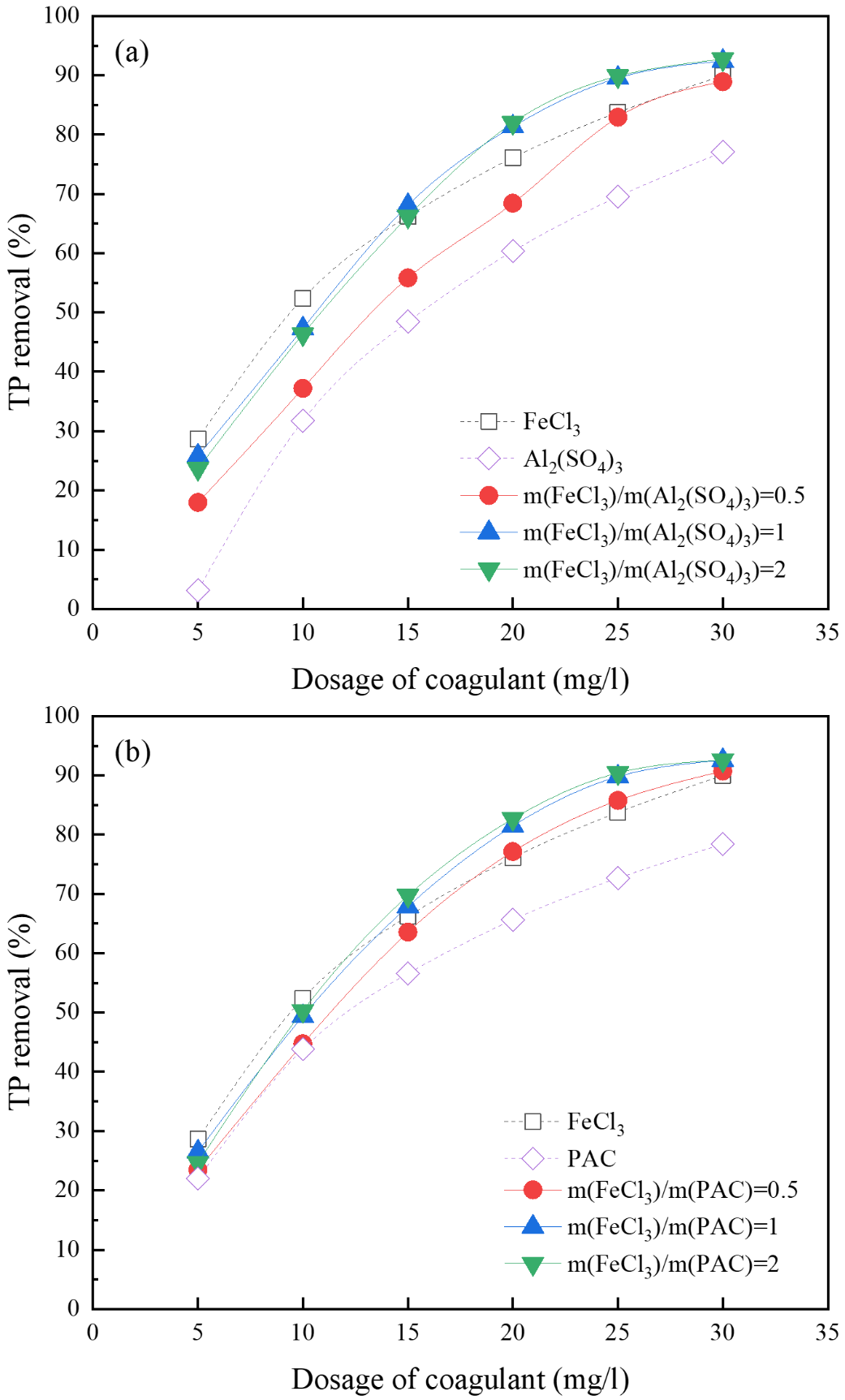


**Fig.SI3** TP removal rate of FeCl_3_-Al_2_(SO_4_)_3_ (a), FeCl_3_-PAC (b) composite coagulants


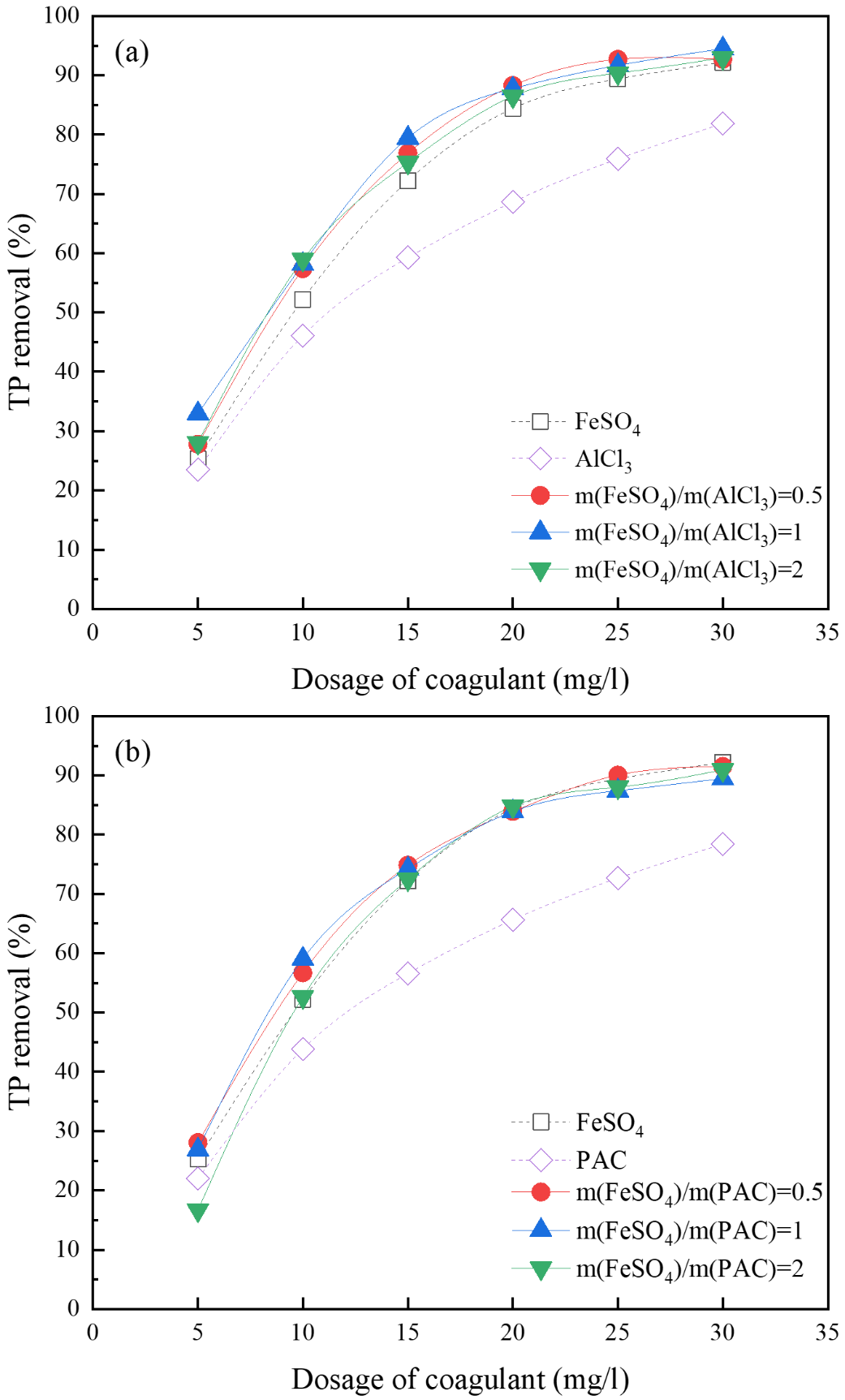


**Fig.SI4** TP removal rate of FeSO_4_-AlCl_3_ (a), FeSO_4_-PAC (b) composite coagulants


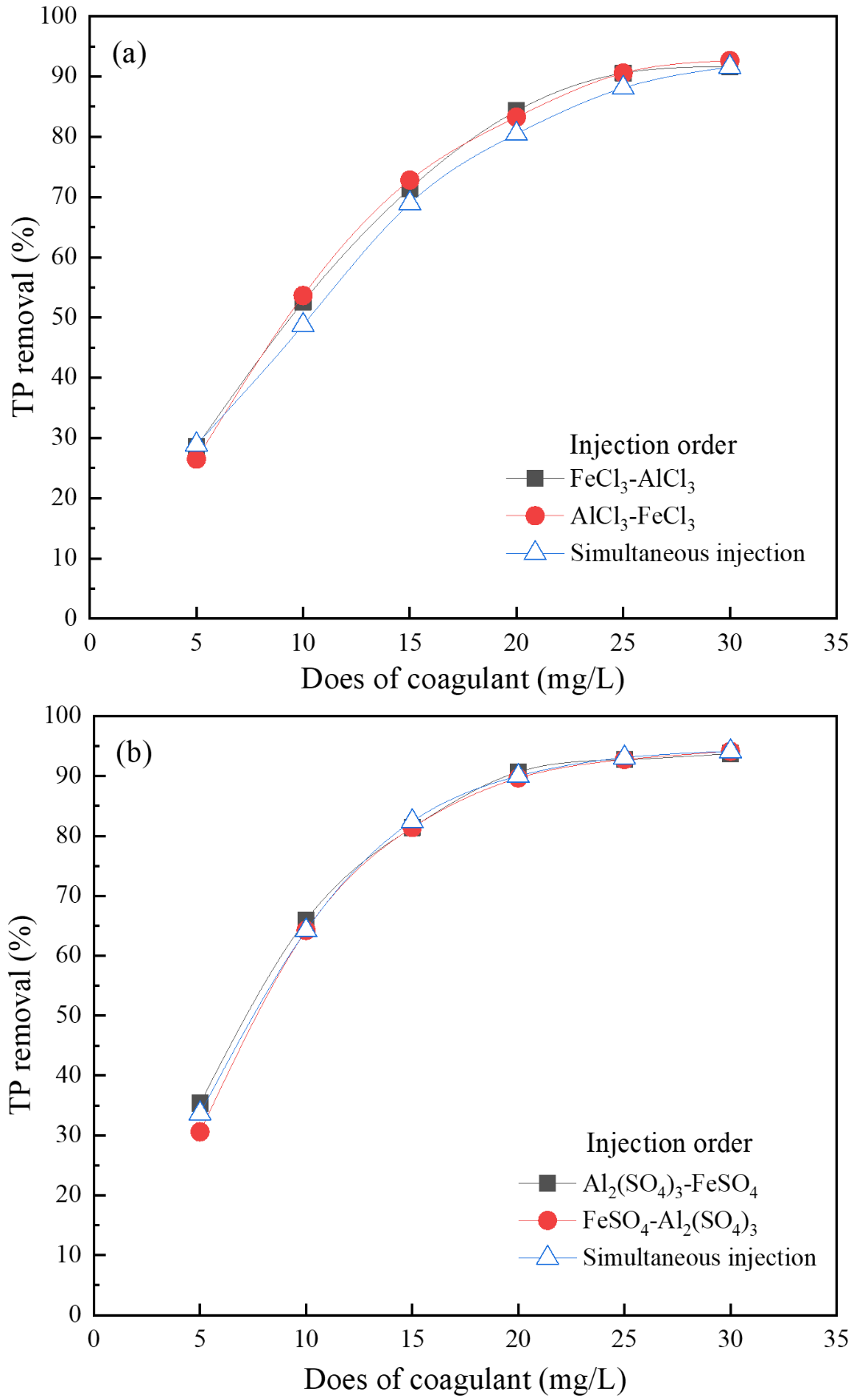


**Fig.SI5** Effect of injection order on the TP removal rate of FeCl_3_-AlCl_3_ (a), FeSO_4_-Al_2_(SO_4_)_3_ (b) composite coagulants

**
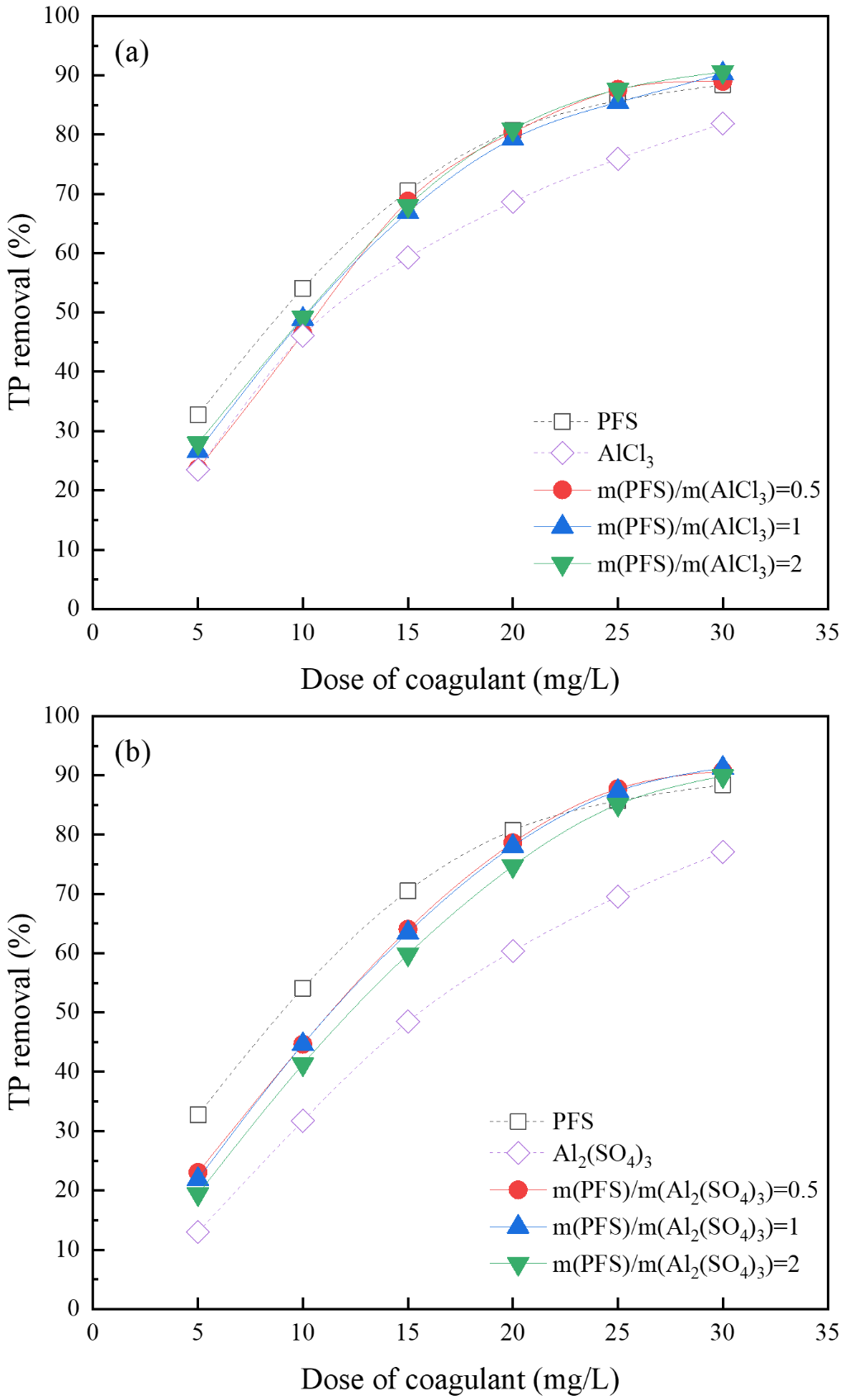
**

**Fig.SI6** TP removal rate of PFS-AlCl_3_ (a), PFS-Al_2_(SO_4_)_3_ (b) composite coagulants


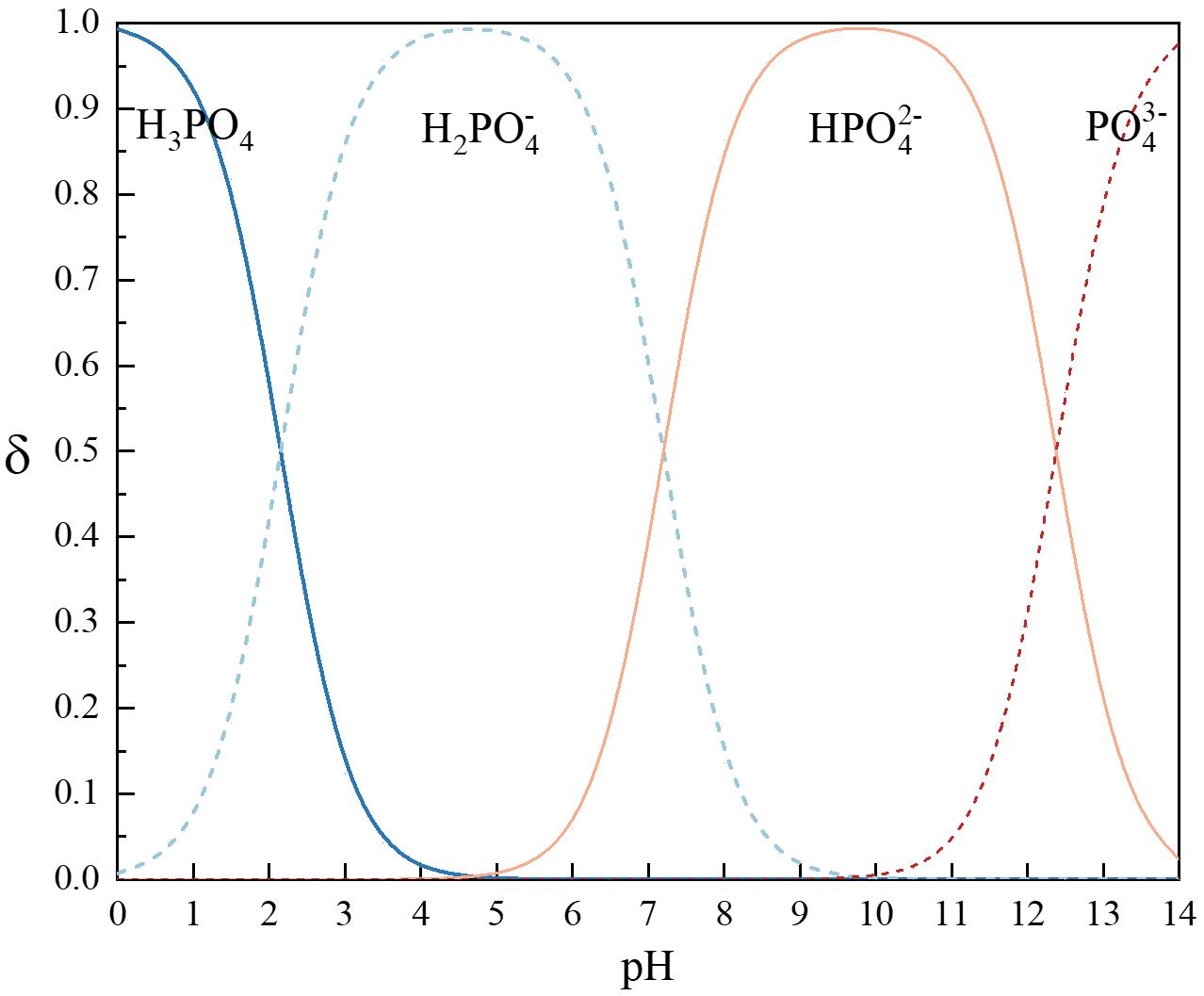


**Fig.SI7** Hydrolysis speciation of phosphate with pH


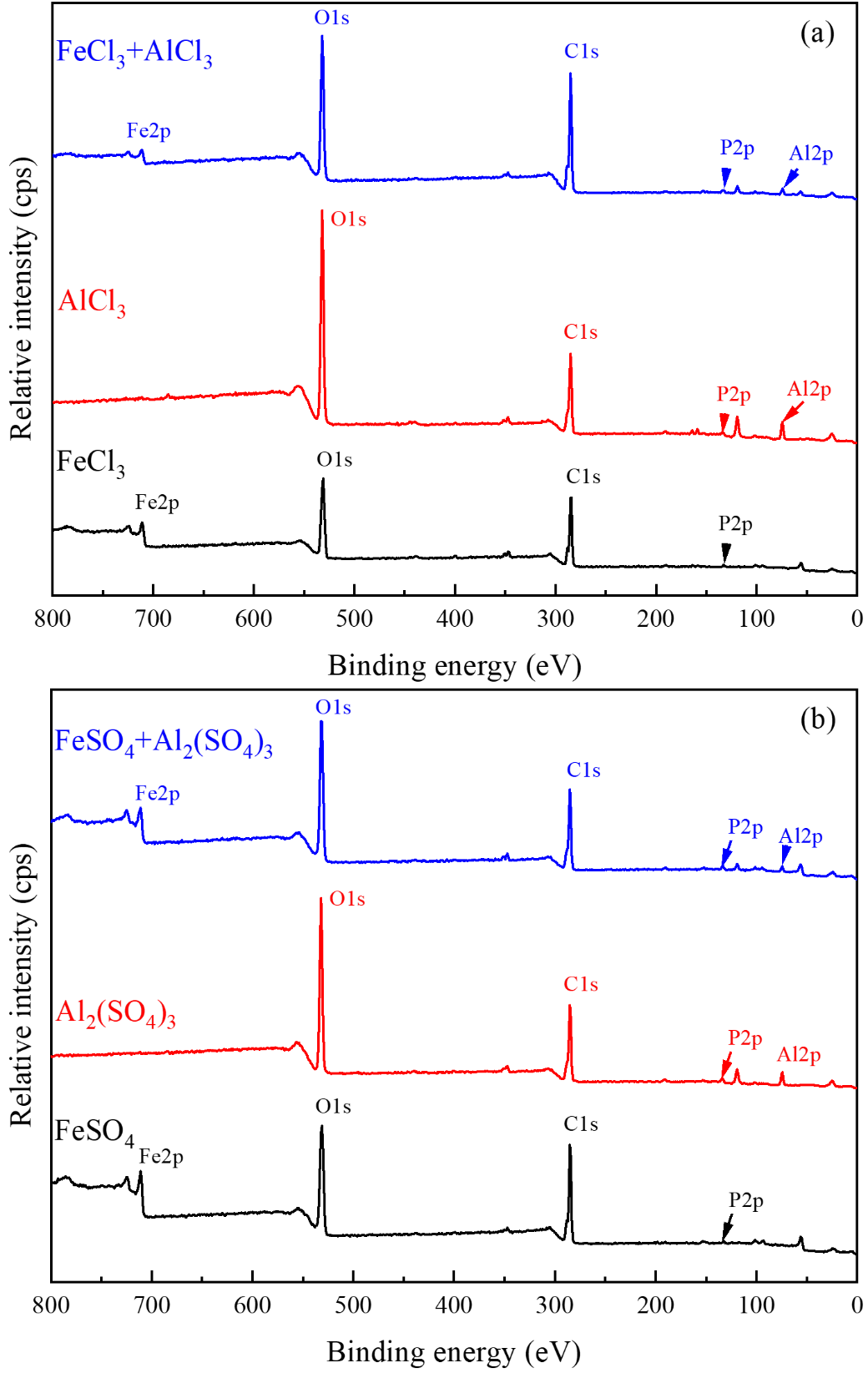


**Fig.SI8** XPS spectra of the precipitates collected in FeCl_3_-AlCl_3_(a), FeSO_4_-Al_2_(SO_4_)_3_(b) composite coagulants


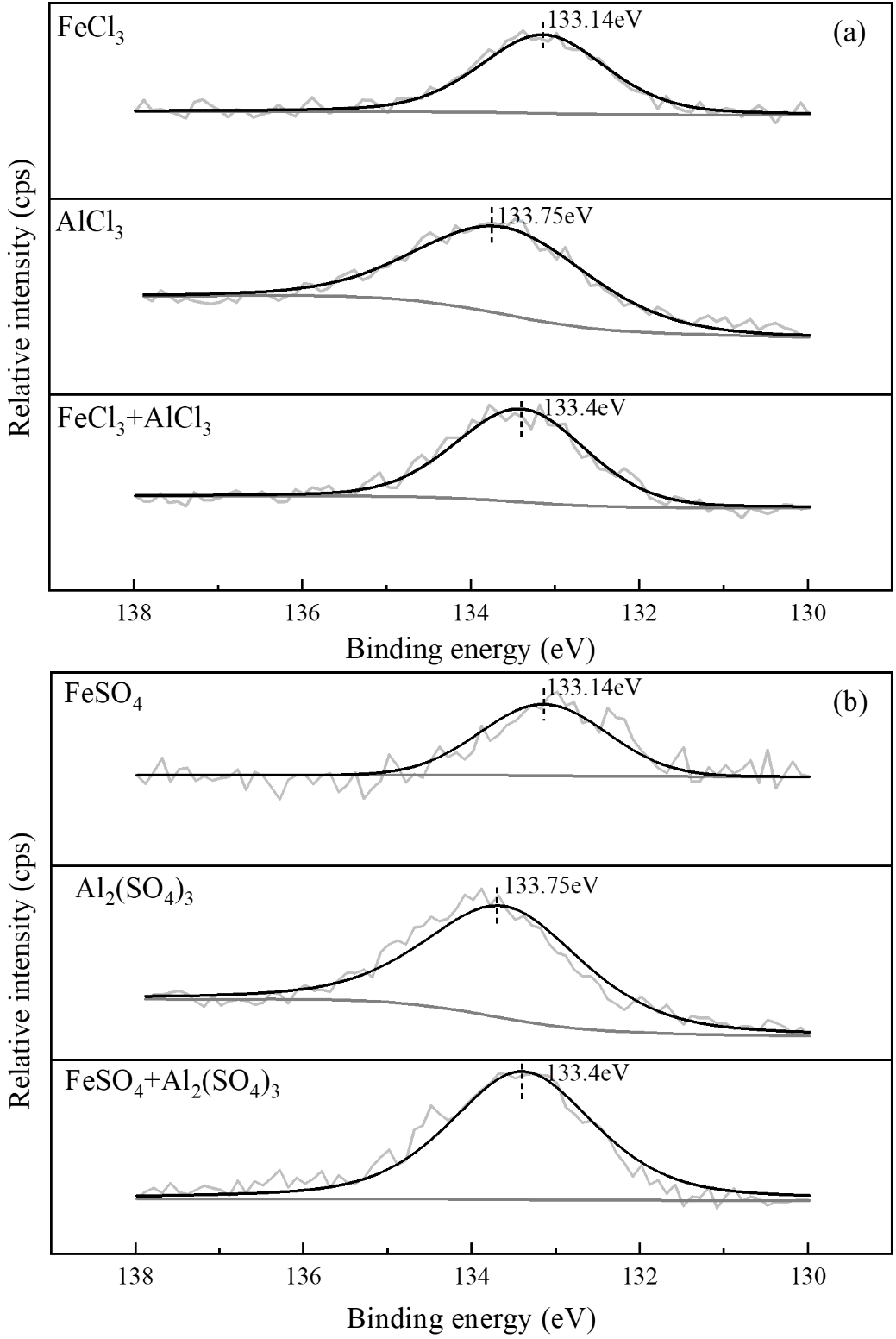


**Fig.SI9** P2p XPS spectra of the precipitates collected in FeCl_3_-AlCl_3_(a), FeSO_4_-Al_2_(SO_4_)_3_(b) composite coagulants
